# Model recommendations meet management reality: implementation and evaluation of a network-informed vaccination effort for endangered Hawaiian monk seals

**DOI:** 10.1098/rspb.2017.1899

**Published:** 2018-01-10

**Authors:** Stacie J. Robinson, Michelle M. Barbieri, Samantha Murphy, Jason D. Baker, Albert L. Harting, Meggan E. Craft, Charles L. Littnan

**Affiliations:** 1 NOAA National Marine Fisheries Service, Pacific Islands Fisheries Science Center, 1845 Wasp Boulevard, Honolulu, HI, USA; 2 University of Washington, Seattle, WA, USA; 3 Harting Biological Consulting, Bozeman, MT, USA; 4 College of Veterinary Medicine, University of Minnesota, St Paul, MN, USA

**Keywords:** Hawaiian monk seal, wildlife disease, vaccination, network model, morbillivirus

## Abstract

Where disease threatens endangered wildlife populations, substantial resources are required for management actions such as vaccination. While network models provide a promising tool for identifying key spreaders and prioritizing efforts to maximize efficiency, population-scale vaccination remains rare, providing few opportunities to evaluate performance of model-informed strategies under realistic scenarios. Because the endangered Hawaiian monk seal could be heavily impacted by disease threats such as morbillivirus, we implemented a prophylactic vaccination programme. We used contact networks to prioritize vaccinating animals with high contact rates. We used dynamic network models to simulate morbillivirus outbreaks under real and idealized vaccination scenarios. We then evaluated the efficacy of model recommendations in this real-world vaccination project. We found that deviating from the model recommendations decreased the efficiency; requiring 44% more vaccinations to achieve a given decrease in outbreak size. However, we gained protection more quickly by vaccinating available animals rather than waiting to encounter priority seals. This work demonstrates the value of network models, but also makes trade-offs clear. If vaccines were limited but time was ample, vaccinating only priority animals would maximize herd protection. However, where time is the limiting factor, vaccinating additional lower-priority animals could more quickly protect the population.

## 1. Introduction

Infectious agents can negatively impact the demographics and fitness of wildlife populations, and disease outbreaks have the potential to threaten the persistence of small populations or endangered species [1,2]. Vaccination has become an important tool for managing disease to protect threatened populations [3]. Network models can help to characterize heterogeneous contact patterns, and are often suggested as useful means of optimizing disease control strategies [4,5]. Network models have demonstrated the potential to maximize vaccination efficiency by targeting those individuals or locations most connected in the network [6,7]. However, we do not know of instances where such model recommendations have been put into practice or evaluated under realistic field conditions encountered during wildlife vaccination efforts. This study provides a novel application of network modelling both to inform and to evaluate a vaccination programme.

The endangered Hawaiian monk seal (*Neomonachus schauinslandi*) could be severely impacted if faced with a disease outbreak. Approximately 1400 monk seals exist as a small and isolated population solely inhabiting the Hawaiian Archipelago [8]. Previous research has demonstrated that Hawaiian monk seals exhibit little genetic diversity [9] and are immunologically naive to many pathogens (including morbillivirus) putting them at high risk in the face of introduced pathogens [10]. Further, the movements and habitats of monk seals in the human-populated islands of the Archipelago put individuals in contact with potential infection sources from anthropogenic impacts, domestic species and other marine mammals [10,11]. Morbillivirus is one pathogen threatening Hawaiian monk seals, and a great concern in marine mammal conservation more broadly. Morbilliviruses, specifically canine distemper virus (CDV), phocine distemper virus (PDV), and cetacean morbilliviruses (CeMV), have long been detected in and associated with marine mammal mortality events [12] and are emerging as a significant mortality source [13]. Morbillivirus was suggested as a potential agent in a major die-off that imperiled the closely related Mediterranean monk seal (*Monachus monachus*) [14]. Because infection is typically spread through aerosolized respiratory droplets, the potential for spread is high even with casual contact or close proximity between individuals [15].

Some vaccines have been adapted for wildlife use and provide disease management options that have benefitted species of high conservation value (e.g., black footed ferrets in the United States [16], Ethiopian wolves in Ethiopia [17]). Vaccination can be particularly effective against morbilliviruses (for example measles [18], and rinderpest [19]). A monovalent recombinant CDV vaccine, commercially produced for use in ferrets (Purevax, Merial Inc., Duluth, GA, USA), has been effective for vaccinating other carnivores including seals [20] and provides an option for protecting Hawaiian monk seals from a morbillivirus outbreak. Vaccinating free-ranging wildlife is a difficult undertaking, and there are many considerations in deciding how to best deploy the vaccine [7,21,22]. Extensive epidemiological modelling has demonstrated the need for prophylactic vaccination of Hawaiian monk seals against morbillivirus [23]. But, the question remained: how to most efficiently use this vaccine to protect the population.

The heterogeneity and configuration of contacts between individuals are critical in shaping epidemic parameters such as rates of spread and epizootic size, making network models a valuable tool in understanding disease dynamics and planning interventions [24–26]. Network analysis is often suggested as a useful tool in prioritizing vaccination efforts by targeting the individuals with the highest contact rates or probability of spreading disease [4,5,27]. Network metrics of connectivity provide a particularly good guide for targeting disease interventions because they relate well to time-to-infection and overall risk [28]. Several studies have used social network analysis and network simulation models to suggest a targeted approach in disease management, including studies of agricultural systems [22] as well as wildlife populations [29,30]. Targeting the most connected individuals in the network substantially increases efficiency in simulation studies, requiring fewer vaccinations to decrease transmission [6]. Yet, moving from network models to designing interventions remains one of the great challenges in this growing field of epidemiological research [31].

Our goal was to use network analysis to identify the key seals to vaccinate based on contact patterns. To this end, we used behavioural observations and seal sightings to construct contact networks. We then used the descriptive statistics from these empirical networks to guide our strategy and vaccinated a major component of the Hawaiian monk seal population on the island of Oahu. But, the implementation of the vaccination programme brought logistical constraints and field conditions beyond the model's scope. Thus, we had a unique opportunity: to test the efficacy of model recommendations applied to reality. For this purpose, we used dynamic network models to simulate epizootics and evaluate the efficiency of in-field reality compared to the ideal vaccinations we had strategized.

## 2. Material and methods

### (a) Study area and population

**Figure 1.**
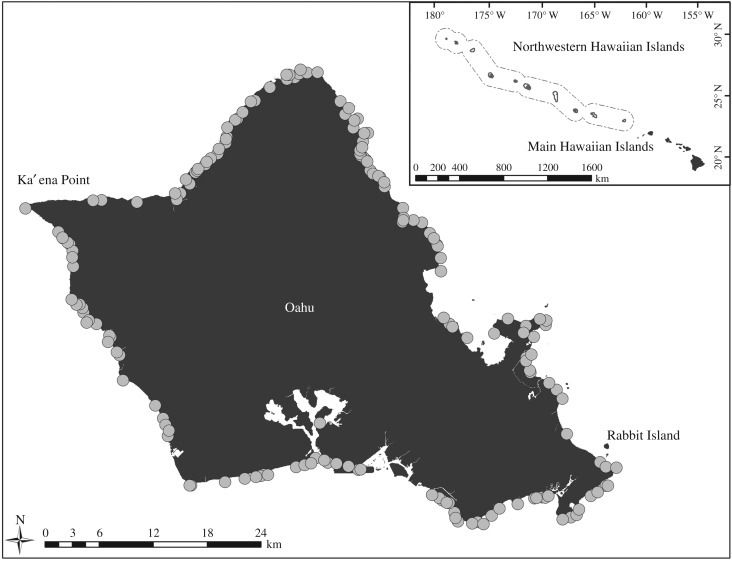
A map of Oahu shows beaches with Hawaiian monk seal sightings reported in 2015 (grey circles). Areas of intensive behavioural observations are labelled. Inset shows the species range.

The endangered Hawaiian monk seal exists (solely) throughout the Hawaiian Archipelago, including the human-inhabited ‘main’ Hawaiian Islands (MHI), as well as the smaller remote islands and atolls making up the Northwestern Hawaiian Islands (NWHI) (figure 1). The majority of the population (about 1100 seals) is in the NWHI, while about 300 animals inhabit the MHI [8]. Given the long-time geographical isolation and genetic similarity across the species' range [9], all seals are considered to be similarly susceptible to morbillivirus or other disease outbreaks. While MHI and NWHI seals bear similar risk of exposure to PDV or CeMV from other marine mammal species in Hawaiian waters, MHI animals bear the additional risk of exposure to CDV from domestic dogs. This project focused on the MHI subpopulation, as mixing between NWHI and MHI subpopulations is uncommon on timescales relevant to morbillivirus spread [32]. Monk seal subpopulations on each island of the MHI are small (tens of individuals versus more than 200 seals at some NWHI sites), making it possible to achieve herd immunity with modest numbers of animals vaccinated. Additionally, the MHI are more accessible, with National Oceanographic and Atmospheric Administration (NOAA) staff and veterinarians available to monitor animals throughout this effort. This research served as a pilot project upon which future effort will be expanded to achieve herd immunity throughout the species range.

Specifically, the island of Oahu was the target of the pilot vaccination programme. The island of Oahu contained 365 km of shoreline and over 100 beaches used by 44 monk seals in 2015. The primary source of data regarding seal sightings and locations came from an extensive volunteer network that regularly monitored beaches and collected publicly-reported sightings through a hotline. Data from each seal sighting (seal identification, location, day/time) were stored in a database maintained by NOAA.

### (b) Analytical approach

**Figure 2.**
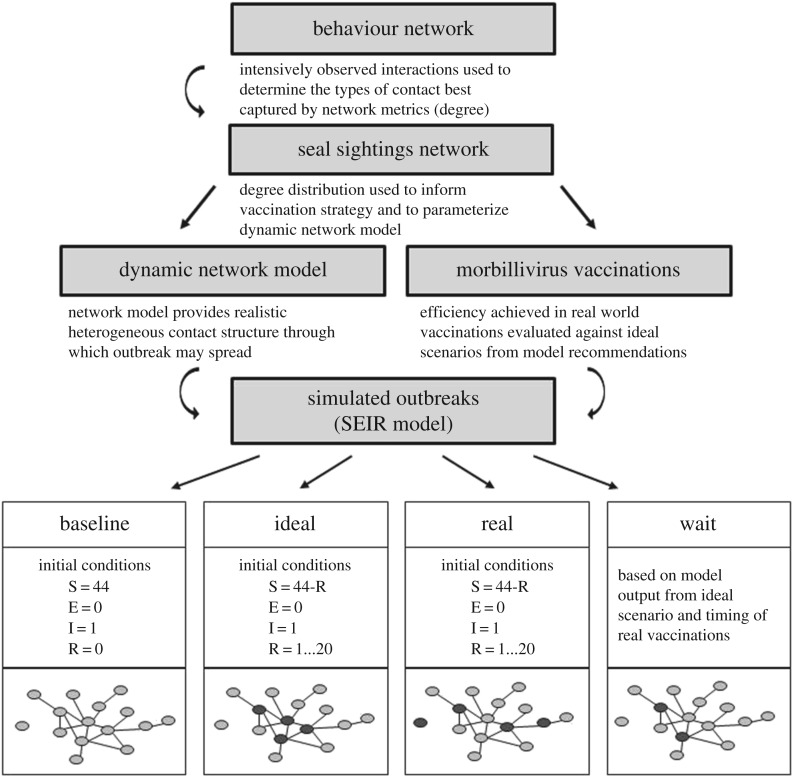
Schematic shows how each step of network analysis builds on the other and describes the outbreak scenarios simulated over the dynamic network model. SEIR, susceptible-exposed-infectious-removed; dark circles, vaccinated seals.

We used a multi-step approach applying network analysis (figure 2). First, we constructed a descriptive ‘behaviour network’ based on intensive behavioural observations of a population subset to determine how well the network described contact relevant to disease transmission processes. We then built a larger descriptive ‘seal sightings network’ based on less intensive, more comprehensive sightings data for all Oahu seals in 2015. From the seal sightings network, we calculated network statistics both to inform the strategy for vaccinating monk seals against morbillivirus and to parameterize a model to evaluate efficiency of the vaccination effort. Finally, we constructed a ‘dynamic network model’ over which epizootics were simulated. We used the extent of simulated outbreaks to measure the efficiency of vaccination scenarios.

### (c) Behaviour network: evaluate network fit to behavioural observation

As a preliminary step in understanding the types of behaviour and potential for pathogen-transmitting contact, we conducted intensive observations of seal behaviour on two Oahu beaches with typical monk seal habitat, Rabbit Island and Ka'ena Point, where numerous seals were known to come ashore (figure 1; see the electronic supplementary material for details). These observations were used to add context to the coarser interaction data available in the seal sightings database. We characterized types of seal contact, including proximity at specified distances (less than 5 m, 5–10 m, 10–20 m, 20–50 m) and direct interactions (including gentle behaviours like nudging and aggressive behaviours like playing or fighting).

We constructed a contact network based on observed associations between seals. We treated each seal as a node (point) and each interaction (of any type) as an edge (line) in the network created using the ‘igraph’ package [33] in program R [34]. As a preliminary analysis, we calculated statistics for each node (degree, eigenvector centrality, coreness, betweenness, closeness, transitivity) to determine which types of interaction were best represented by which network statistics (details in the electronic supplementary material).

### (d) Seal sightings network: construct population-wide contact network

A more complete network of seals on Oahu was necessary to assess population-wide heterogeneity of contact rates to inform vaccination strategies. Based on the information from the behaviour network (see the electronic supplementary material, results), we determined that seals seen on the same beach in the same day most likely came into close proximity or direct contact at some point during the onshore session, thus providing sufficient contact for transmission of morbillivirus. We queried the NOAA seal sightings database for all sightings on Oahu in 2015 (to plan the 2016 vaccination effort). Preliminary investigations of sightings from each month revealed no seasonal variation suggesting the system was well-represented by the full year's data (electronic supplementary material, figure S2 and table S3). We treated each seal as a node and created edges for each report of two seals sharing the same beach in a given day (using ‘igraph’ in R). We calculated degree for each node and used this statistic to rank seals from the most to least connected. Degree performs well in capturing how contact heterogeneity impacts disease spread [28]. We prioritized those with the highest connectivity (highest contact rates) for vaccination. Additionally, we used the degree distribution observed in this empirical network to inform the parameters of the dynamic network model in our next step.

### (e) Dynamic network model: construct a model based on empirical network statistics

Based on the contact structure observed in the island-wide seal sightings network, we constructed a dynamic network model. We used the R package ‘EpiModel’ [35], which uses degree statistics from an observed network to parametrize a model that simulates the process of the contacts forming and dissolving across the network at each time step in a simulated epidemic. Disease dynamics can then be simulated across this dynamic contact network. The ability to simulate disease spread over a dynamic network in EpiModel overcomes one of the long-standing limitations in modelling disease processes where networks are treated as static [25,31].

Scaling network models appropriately to pathogen-specific transmission attributes and infectious periods is important to reflect the number of contacts occurring in a timespan relevant to the disease process [25,36]. We used a daily time step in the model to allow multiple steps within the two-week infectious period of morbillivirus. Because the observed network was based on a full year of observations, the number of contacts had to be rescaled to reflect the probability of contacting another individual on a daily basis. Briefly, based on the frequency of seal sightings across Oahu, we considered a week of effort to capture a single full-island snapshot constituting one model time step (see the electronic supplementary material for details).

To represent the heterogeneity in contact within the Oahu monk seal population, we assigned the nodes in the dynamic network model contact rates based on the degree distribution of the seal sightings network. The 45 nodes (representing the 44 observed monk seals plus one infectious seal) of the dynamic network model were split into nine sets of five (because specifying a target degree for each node would over-fit the model) where set 1 was assigned the average degree of the five seals with lowest degrees in the seal sightings network, set 9 was assigned average degree of the five seals with the highest degrees, etc. (table 1). Node sets did not influence which nodes could come into contact (assortivity parameter = 0). Because it is typical for a seal to rest onshore in one area for a portion of a day and then use a different area another day, we set the contact duration to allow contacts to form and dissolve at each time step (duration parameter = 1 day).

**Table 1.** Degree distribution is summarized in ranked node sets. (Seal sightings network (SSN) shows the observed data, which were then rescaled^a^ for use as target values to parametrize the dynamic network model (DNM).)

node set (5 each)	SSN mean degree	degree target values (SSN rescaled)	DNM output degree (mean of simulations)
total no. of edges	221.00	4.25	4.30
overall	9.85	0.19	0.19
set 1	0.25	0.00	0.00
set 2	3.00	0.06	0.07
set 3	5.00	0.10	0.11
set 4	7.60	0.15	0.15
set 5	10.40	0.20	0.20
set 6	12.00	0.23	0.23
set 7	13.80	0.27	0.26
set 8	15.60	0.30	0.30
set 9	21.00	0.40	0.41

^a^Degree statistics from the sightings network based on a full year of sightings were down-scaled for use with daily time steps in the dynamic network model (see the electronic supplementary material for details).

### (f) Vaccinate free-ranging Hawaiian monk seals

Seals identified by the seal sightings network as having high contact tendencies (i.e. potential key disease spreaders, based on degree) were given high priority for vaccination. However, other factors also impacted priority, as any seal with a suspected health concern was avoided. Further, though we did not anticipate any difference in response between males and females, as an extra caution with an endangered species, reproductive females were avoided during this pilot stage of the vaccination effort. In addition to these limitations on vaccination candidates, logistics also played a role in which individuals received vaccinations. A seal had to be on an accessible beach and in a position allowing safe approach.

The initial vaccination effort for wild Hawaiian monk seals began on Oahu in February 2016. Sufficient doses were available to vaccinate all Oahu monk seals; however, expiration dates ranged from April to October 2016. Vaccination efforts ended in October 2016, prior to the expiration date of available vaccines. Our goal, therefore, was to vaccinate a sufficient number of animals to achieve herd immunity as efficiently as possible. Seals were vaccinated according to protocols developed by NOAA based on data from captive seals (P.K. Yochem 2013, unpublished data; F. Gulland, T. Kendall 2011, unpublished data). In brief, routes of vaccine administration included hand injection and pole syringe injection (Jab Stick, Dan-Inject, Austin, TX, USA). Seals received a booster vaccination three or more weeks after the initial vaccine was administered.

### (g) Evaluate ideal versus real vaccination efficiency with SEIR model on simulated network

Once vaccination efforts were completed, we evaluated how well our network-informed strategy held up in the face of field realities. We used the dynamic network model to represent the seal population through which an epizootic could spread after various vaccination scenarios. We used an SEIR model to model the flow of animals between disease states from susceptible (S), to exposed (E), to infectious (I), to removed (R). The state ‘R’ may encompass any animals removed from the susceptible pool whether through post-infection immunity, vaccination-induced immunity, or disease-induced mortality. Given the severity of morbillivirus outbreaks in other marine mammal species, we assume that most seals would die rather than recover from infection. We parametrized the SEIR model based on previous epidemiological modelling in which Baker *et al*. [23] simulated a wide range of scenarios to investigate the potential impacts of a morbillivirus outbreak in monk seals. To isolate and thus evaluate the role of contact heterogeneity in the impact achieved by our vaccination efforts, we based our model parameters on the worst case values of the Baker *et al*. [23] model (i.e. those that would most favour disease transmission and outbreak perpetuation). Therefore, we set disease transmissibility at 1.0, and the latency period (time spent in the ‘exposed’ compartment) and infectious time (time in ‘infected’ compartment) were each 14 days (electronic supplementary material, table S4, and see the electronic supplementary material for trials with other parameter values). We varied only which seals were vaccinated (i.e. which nodes were initialized in the ‘removed’ state before the simulated outbreak).

We evaluated the efficiency of our vaccination effort based on the number of nodes infected at the end of each simulated outbreak after a given level of vaccination. We ran three different model scenarios. The ‘Baseline’ scenario was initialized with 44 nodes susceptible, a single node infected, and no vaccinations given. For the ‘Ideal’ scenario, the highest contact seals (high degree nodes) were vaccinated (designated as removed, ‘R’, at model initialization, repeated for 1 … 20 vaccinations mimicking numbers vaccinated in reality). For the ‘Real’ scenario, nodes were initialized as ‘R’ according to degree corresponding with contact rate of seals in the order actually vaccinated, i.e. if the first seal vaccinated had a low degree according to the seal sighting network, a low-degree node in the dynamic network model would be classified as ‘R’ before running the SEIR model (repeated for 1 … 20 vaccinations). We ran each model for 1000 simulations over 100 (daily) time steps (sufficient time for epidemics to run their course in preliminary trials) at which nodes came into contact according to contact structure established by the dynamic network model, and individuals became exposed, infected, or removed according to parameters of the SEIR model (electronic supplementary material, table S4).

The Real scenario differs from the Ideal for practical reasons. For example, we did not always find the ideal seal at the ideal time. We created a fourth scenario based on the outputs of the Real and Ideal models. Labelling this the ‘Wait’ scenario, we supposed that non-priority seals were passed up while waiting to vaccinate only the top priority seals (those with the 10 highest degrees). In the Wait scenario, we assigned each level of vaccination the outcomes from the Ideal model, but the level of vaccination was only increased when priority seals were vaccinated (i.e. if the first six seals vaccinated only included two priority seals, the Real scenario would show the impact of all six vaccinations, while the Wait scenario would only show the impact of two priority vaccinations). This scenario allowed us to determine the best approach if time or vaccine supplies were more limiting.

## 3. Results

### (a) Behaviour network: evaluate network fit to behavioural observation

A total of 14 individual seals (32% of the island-wide population) were observed on Oahu. Seventy-eight per cent of all observed seal associations (*n* = 825) were based on proximity alone (no body contact between individuals occurred). The remaining 22% of associations involved direct contact (14.5% involved gentle interactions, 7.5% were aggressive interactions).

Seal interactions based on proximity or gentle interactions were well represented in the contact network (electronic supplementary material, figure S1), and degree was strongly correlated with observed levels of interaction (electronic supplementary material, table S1). Conversely, none of the network statistics correlated with the time spent in aggressive interactions, indicating that seals involved in fight or play might seek out such interactions regardless of the number of other seals with which they casually interact. However, contact defined by shared beach location represented behaviours relevant to spreading diseases, such as morbillivirus, that require only close proximity for transfer of aerosol particles.

### (b) Seal sightings network: construct population-wide contact network

**Figure 3.**
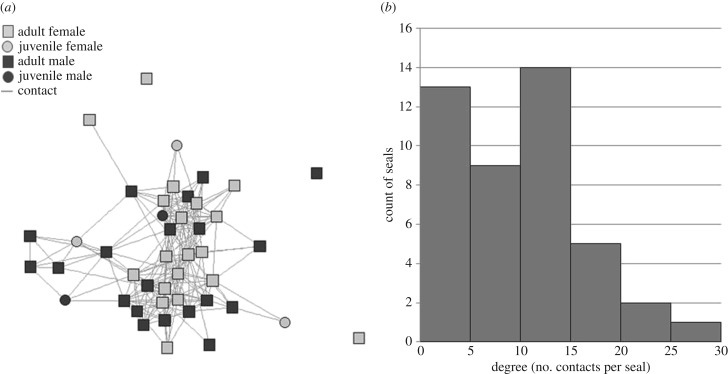
(*a*) Network structure and (*b*) degree distribution illustrate the heterogeneity in contact rates among Oahu's monk seals.

The seal sightings network based on all Oahu seal sightings for 2015 (*n* = 2540 sightings, 44 seals) showed high connectivity between seals, with heterogeneity in contact patterns (figure 3). Mean distance between nodes was 1.96, indicating fewer than two intermediate contacts connecting two average seals. The network's cohesive structure (figure 3*a*) indicated a lack of key individuals whose removal could fully dissolve the network. The mean degree of the network was 9.85, but nearly half of nodes (21 seals) had 10 or fewer links in the network, whereas the five most connected seals accounted for 24% of the total links in the network (figure 3*b*). The heterogeneity in degree and lack of distinct clusters suggested that targeting high-degree nodes to make the network sparser would be more effective than targeting a few central nodes to fragment the population into hard-to-reach subgroups [31]. The 10 highest-degree seals were given top priority for vaccination, followed by those ranked 11–20.

### (c) Dynamic network model: construct a model based on empirical network statistics

The dynamic network model provided a close approximation of the contact patterns described by the seal sightings network. After running a 1000 iteration burn-in period, the degree of nodes from the dynamic network model closely matched the target values (based on the degree distribution from the seal sightings network) used to parametrize the model (table 1).

### (d) Vaccinate free-ranging Hawaiian monk seals

During the field effort from February-October 2016, we fully vaccinated 21 wild Hawaiian monk seals on Oahu (table 2). An additional three seals received an initial vaccination, but no booster. The success of this effort required an average of two staff dedicated for field trips to seek out vaccination candidates one day per week. Additionally, staff responded to volunteer reports of candidate seals onshore on accessible beaches. Volunteer reports were particularly relied upon for seals needing boosters since search efficiency greatly diminished when seeking one particular seal. NOAA staff delivered 3–10 vaccinations (initial and booster) per month. While our goal was to booster animals within 3–5 weeks of the initial vaccination, the booster time accomplished ranged from 21 to 79 days (mean = 34 days).

**Table 2.** Hawaiian monk seals vaccinated against morbillivirus on Oahu, Hawaii, in 2016.

	male	female	adult	juvenile	weanling	total
no. seals fully vaccinated:						
	11	10	12	5	4	21^a^
no. seals partially vaccinated:						
	2	1	2	1	0	3^b^

^a^Two seals died from causes unrelated to vaccination.

^b^One seal died from causes unrelated to vaccination prior to booster.

Eight of the top 10 seals on the priority list were vaccinated. The remaining top candidates primarily came ashore on the offshore islet, Rabbit Island. While these seals were sighted regularly during the vaccination effort (via spotting scope from Oahu's mainland), they were never accessible on the days favourable for boating to the islet. Two more seals in the network top 20 remained unvaccinated at the end of the effort: one favoured rocky beach spots and another was not sighted during the 2016 vaccination effort. In addition to seals represented in the original network, four weaned pups were vaccinated that were not part of the population at the time the seal sighting network was constructed.

### (e) Evaluate ideal versus real vaccination efficiency with SEIR model on simulated network

**Figure 4.**
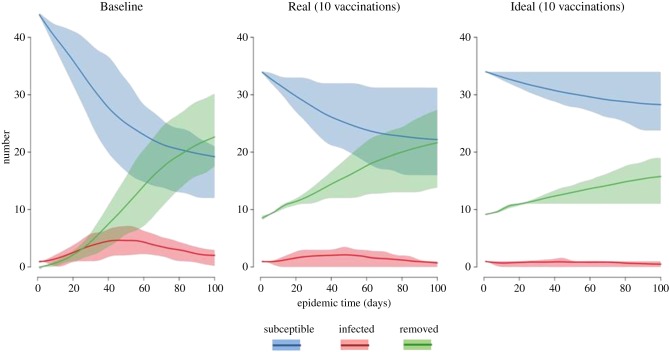
Epidemiological curves show the progression of simulated morbillivirus outbreaks in Hawaiian monk seals after different vaccination scenarios (mean of 1000 simulations with 95% confidence bounds shaded). The ‘exposed’ group was included in models, but not plotted for simplicity of the figure.

The Baseline SEIR model, showing the likely pattern of an epizootic spreading through an unprotected population, resulted in an average of 22.3 infected nodes and an epizootic peaking on day 52 (figure 4). The Real and Ideal scenarios showed similar patterns with epizootics peaking slightly earlier (day 47 and 40, respectively), but the infection rate and total numbers of animals infected decreased in model runs with more nodes ‘removed’ at model initialization (figure 4). In the Real scenario, the first 10 nodes vaccinated reduced the total number infected to 11.2 nodes, whereas in the Ideal scenario after 10 vaccinations there were only 5.3 infected nodes (averaged over 1000 model runs). The differences between Real and Ideal scenarios were minimal in runs after 20 vaccinations, with outbreaks of 2.1 nodes in the Real scenario and 0.9 nodes in the Ideal. The improved efficiency of the Ideal over the Real vaccination scenario was consistent over a wide range of other parameters used in supplemental model trials (electronic supplementary material, figure S3).

**Figure 5.**
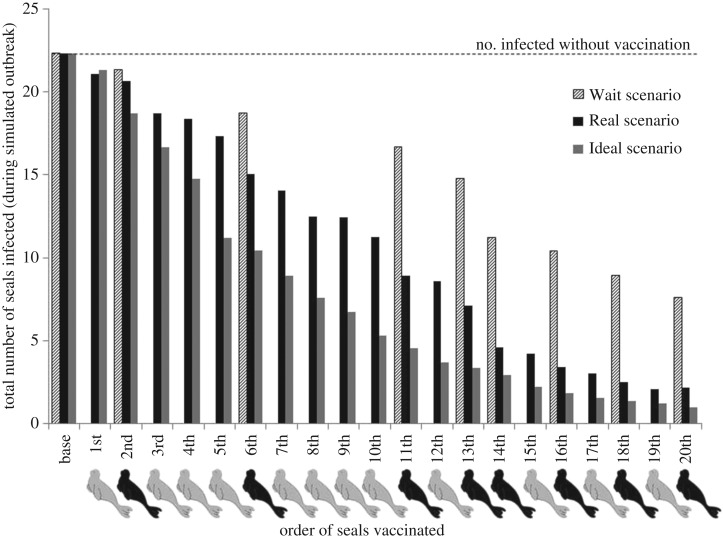
Decrease in numbers of monk seals infected with morbillivirus achieved under different vaccination strategies (Ideal, Real and Wait). The *x*-axis indicates the order in which seals were vaccinated in the Real scenario (black for seals on the top priority list, grey for others). Bars show average number of animals infected in simulated outbreaks assuming disease was introduced after a given number of animals were vaccinated.

In the Ideal scenario, there was a greater decrease in numbers infected with each increase in number vaccinated (figure 5). To achieve a given decrease in numbers infected during an outbreak, the Real scenario required 44% more vaccinations than the Ideal scenario. However, if we consider the timing with which animals were detected, as in the Wait scenario, it became obvious that foregoing vaccination of non-priority seals while waiting for the Ideal vaccination candidates would have produced a slower decrease in numbers infected (figure 5).

## 4. Discussion

This work provides a rare example of widespread effort to vaccinate an endangered marine mammal population. The numbers of monk seals vaccinated in this initial effort are expected to be sufficient to limit the spread of disease, should morbillivirus be soon introduced into the subpopulation on Oahu.

We took advantage of the unique opportunity to assess the effectiveness with which network model recommendations were applied to a real-world disease management programme. Extensive reports of identifiable individuals were a critical component in our ability to construct a full contact network for targeting specific individuals for vaccination. Our application of a rigorous modelling approach, simulating epidemics over a dynamic network based on empirical association data, was instrumental in evaluating the use of the network-recommended targeted vaccination approach. While precisely following network recommendations could decrease disease transmission with the fewest numbers of vaccines, we found that vaccinating extra lower-priority animals let us achieve population protection more quickly than waiting for access to the ideal set of vaccination candidates.

While network analysis has been used to inform vaccination or management strategies [6,22,29,30], we know of no programmes implementing a network-informed vaccination strategy or evaluating such a strategy once implemented. If, as in Rushmore *et al*. [6], we had found contact rates highly correlated with characteristics such as sex or age, it may have made the group-targeted approach more efficient than targeting individuals. Targeting individuals or specific locations may be more practical in different settings where they are more predictably sighted or contained (as in agriculture) [7].

Our behavioural observations add to the understanding of social behaviour and interactions in seal populations and compliment previous work on contact rates of monk seals in the remote NWHI [23]. In one location in the NWHI, where the monk seal subpopulation is much denser than on Oahu (20 seals km^−1^ of coastline versus 0.12 seals km^−1^), Baker *et al*. [23] calculated a network with one large component indicating high contact among all seals in the population. They found heterogeneities by sex and age classes, with sub-adult males showing the highest rates of contact. Our results were partially similar; adult males were more likely to be involved in aggressive interactions, but seals of all age and sex classes were similarly represented in proximity associations. By contrast, Wolf *et al*. [37] studied a colonial sea lion species and found that maternal territories formed the basis of localized contact structure.

We must acknowledge potential limitations or complications with our analysis. We know that our contact network, while it probably included all seals on Oahu during the observation period, cannot possibly capture every contact. Not all beaches were surveyed every week, and we did not observe seal interactions at night or in water. We know that missing contacts can be problematic and bias network results [38]. However, given that all seals spend part of their time out to sea and are not always observed, we do not expect systematic bias in missing contacts, making the relative contact rates representative though not numerically exact. Additionally, because we defined the study area as the entire island of Oahu, we avoided drawing artificial boundaries, which can have more serious implications than missing contacts of the nodes included in the network [38].

Animal movement complicates disease management [39–41]. Though Oahu is an island, Oahu monk seals are not a truly closed population. Over the course of our study, pups were born and animals died. Four of the animals vaccinated were weaned pups not included in the original network based on 2015 observations, and three animals died of causes unrelated to vaccination after they were vaccinated (table 2). Yet, we did not incorporate demographic processes into the simulation models as this was beyond the primary goal of the analysis and unlikely to have substantial impact in the short time of the simulated outbreaks. Vaccination efforts of sufficient coverage can still be (and have been) effective in unbounded populations, both in terms of cost-effectiveness [42] and effectively halting disease spread [43].

The current analysis evaluated *efficiency* of our efforts to vaccinate sufficient numbers of individuals to interrupt potential chains of pathogen transmission. We do not have the data to assess *efficacy* of the vaccinations, as disease exposure trials would not be ethical with an endangered animal. However, previous studies in captive animals showed that Hawaiian monk seals mount a sufficient antibody response after vaccination with the product used in this study (P.K. Yochem 2013, unpublished data; F. Gulland, T. Kendall 2011, unpublished data). Seroconversion has also been documented in other marine mammals [20,44], and veterinary trials have demonstrated antibody response which is protective against CDV infection in domestic species [45].

Through our research, we learned that despite the best data availability and modelling efforts to inform a management strategy, with reality comes trade-offs. Here, we saw that going against the network-recommended priority vaccination targets required more vaccinations to achieve a given level of immunity in the Oahu subpopulation; however, we were able to administer more vaccines to non-priority animals in a more time-efficient manner than restricting efforts to only specified animals. The network-informed strategy would be important for maximizing probability of achieving herd immunity if faced with limited vaccine doses. However, if time is the more constraining factor, vaccinating available animals may be the quickest route to herd immunity (or other desired management outcome). Future vaccination efforts for the Hawaiian monk seal species will probably focus on working most time-efficiently to build herd immunity in as many segments of the population as possible. Vaccination efforts for Hawaiian monk seals are expected to continue, pending vaccine availability and will build on this pilot effort to protect the remaining subpopulations across the species' range and eventually shift to a maintenance phase, where vaccination efforts specifically target new, susceptible members of the population (births).

## Supplementary Material

Supplemental Information

## Data accessibility

Data are available through the NOAA/NMFS/Pacific Island Fisheries Science Center data repository at inport.nmfs.noaa.gov.
