# Supplementary material for: Model recommendations meet management reality: implementation and evaluation of a network-informed vaccination effort for endangered Hawaiian monk seals
Source: Proc Biol Sci. 2018 Jan 10;285(1870):20171899. doi: 10.1098/rspb.2017.1899 (PMC5784189; doi:10.1098/rspb.2017.1899)
Supplement: Supplemental Information [file rspb20171899supp1.pdf]

TITLE: Model recommendations meet management reality: Implementation and evaluation of a network-informed vaccination effort for endangered Hawaiian monk seals: Supplemental Information

AUTHOR DETAILS:
Corresponding Author:
Stacie J. Robinson, NOAA National Marine Fisheries Service, Pacific Islands Fisheries Science Center, 1845 Wasp Blvd, Honolulu, HI
phone: 808-725-0541
email: Stacie.Robinson@noaa.gov

Michelle M. Barbieri, NOAA National Marine Fisheries Service, Pacific Islands Fisheries Science Center, Honolulu, HI
Samantha Murphy, University of Washington, Seattle, WA
Jason D. Baker, NOAA National Marine Fisheries Service, Pacific Islands Fisheries Science Center, Honolulu, HI
Albert L. Harting, Harting Biological Consulting, Bozeman, MT
Meggan E. Craft, College of Veterinary Medicine, University of Minnesota, St Paul, MN
Charles L. Littnan, NOAA National Marine Fisheries Service, Pacific Islands Fisheries Science Center, Honolulu, HI

Supplemental Sections

Details of intensive behavioral observations used in Behavior Network	SI2
Details of Sightings Network Analysis	SI 7
Details of parameterization for Dynamic Network Model	SI 10
Details of parameterization for SEIR Model	SI 11
Supplemental References	SI 15

Details of intensive behavioral observations used in Behavior Network

Methodology

While volunteer sightings reports were collected across Oahu and covered the full seal population, the time spent viewing a given beach or seal may be short, and these sightings did not involve systematic recording of behavior or interactions among animals. In order to understand the types of behavior and potential for pathogen-transmitting contact, we conducted intensive observations of seal behavior. Behavioral observations were focused on 2 Oahu beaches where numerous seals were known to come ashore (to maximize observations of interactions): Rabbit Island, a small uninhabited island with a sand and rock beach off the east shore of Oahu, and Ka'ena Point, a rock and tide pool beach at the far northwest tip of Oahu (labeled in Figure 1).

Each site was visited twice per week for three-hour observation periods from June – August 2015. Seals were individually identified based on hind flipper tags, applied bleach marks, or natural markings such as scars. If identification of some individuals was not possible, characteristics such as age class and sex were still noted. Ethological data were recorded by two observers, so that they could verify seal identification and effectively observe the full length of the beach. Observers used a combination of temporal scan sampling and recording of focal behaviors [as described in 1]. For temporal scans, an observer visually scanned the beach every 10 minutes and recorded the position and behaviors of all seals. Associations between all pairs of seals were noted, whether the seals were associated by any form of directed behavior or were merely sleeping a specified distance apart. Focal behaviors included any interaction between seals (vocalizing, approaching, making direct contact); these were recorded at any time they were observed.

All data collected over the study period were compiled and summarized for each individual. We developed an association index to identify key players in social interactions. Interactions were defined either as:

proximity - seals associated by physical proximity only (distances classified in bins, <5m, 5-10m, 10-20m, 20-50m);

indirect interactions- distant interactions such as vocalizing or approaching without reaching another seal;

gentle interactions - included touching, nudging, and other behaviors that brought individuals into direct contact, but were not likely transfer of bodily fluids;

aggressive interactions - behaviors with a high likelihood of resulting in transfer of bodily fluids including playing, biting or fighting, or sneezing within close proximity of other seals.

Individual seal summaries included total interactions with any other associated seal in the following ways: minutes spent at each proximity interval, minutes observed participating in either gentle or aggressive interactions; total counts of each documented interaction type; total number of interactions observed (tally of how many different seals they associated with each day, totaled over the study period); total minutes observed; and total number of days observed. Duration of interactions refers to the total amount of time individuals spent displaying each behavior. Interactions by count refers to the number of

times each behavior was noted in observational notes. In order to standardize the data between all seals, results for each individual were divided by total number of minutes observed throughout study. The resulting summary statistics include: proportion of time spent associating with other seals at each proximity interval, and proportion of time (minutes and counts calculated separately) spent directly interacting with other seals (both gentle and saliva-transfer interactions). Average number of interactions with other seals per day was also calculated for each individual by dividing total number of observed interactions by number of days seen in the field.

We constructed a contact network in which each of these interactions served as an edge (line) linking the seals (nodes) involved (the Behavior Network described in main text). We used the "igraph" package [2] in R software [3] to calculate the following network statistics to describe the position and connectivity of each node [4]. We calculated correlation coefficients between the network statistics and the behavior summary statistics were calculated to establish the strength of the relationship between the two methods of analysis.

- **Degree** refers to the number of connections an animal has within the network.
- **Betweenness** shows how often an individual is in the path of two separate individuals' connection.
- **Closeness** measures how many steps are required to access every other individual in the network from a given individual.
- **Eigenvector centrality** (EVC) is a ranking measure of an individual's relevance within the network based on the series of links between them and other population members.
- **Coreness** is a measure of how the individual is connected with respect to multiple sub-parts of the network.
- **Transitivity** is a local clustering index which measures the probability that adjacent individuals connected to the given individual are also connected to each other.

The seal summary statistics and network statistics were also compared between sexes to determine if any subset of the population was significantly more likely to act as vectors of disease (age difference were not tested, because few juvenile seals were observed). T-tests were calculated to test for significant differences between male and female monk seals' interaction rates or network statistics.

Results & Discussion

In total, 14 individual seals (female n=6; male n=8; 32% of the island-wide population) were observed across 18 three-hour observational sessions at Ka'ena Point (11 sessions) and Rabbit Island (7 sessions). Of the females observed, there was one adult, three subadults, one juvenile, and one weaned pup. Of the males observed, there were five adults, two subadults, and one juvenile. On an average day, observers found three seals present, though total number of seals noted at either location ranged from 0-5.

We found that network statistics describing the direct connectivity of a given node (degree, EVC, coreness) best related to the indices of observed behavioral interactions

between individuals, whereas metrics describing more complex/path-oriented patterns (betweenness, closeness, transitivity) were not significantly correlated to observed seal interactions (Table SI1). Measures of degree, EVC, and coreness were closely correlated to one another (Pearson's r =0.83 - 0.89) and all were closely correlated to the number of direct interactions for an individual seal (particularly to the time spent in gentle interactions). Conversely, none of the network metrics correlated with the time spent in aggressive interactions (fight or play).

There was tendency for adult males to engage in aggressive interactions more frequently than females (p=0.077) or juvenile males (p=0.015), largely driven by tendency to fight with one another (Table SI2). This could indicate a strong role of males in the transmission of disease, especially for pathogens for which exposure to infectious bodily fluids (blood or saliva from bites) is essential for transmission. However, for pathogens that do not require direct contact for transmission (e.g., aerosolized droplets for morbilliviruses), there was no evidence for a dominant spreader group, as all sex and age classes were equally likely to be in close proximity or have gentle direct contact with others. Interestingly, this strong contact by males is not captured well by the network metrics, likely because we included all forms of contact as links and aggressive contacts are rare, so that network statistics are more representative of more prevalent proximity/gentle contacts.

Table SI1: Correlation between seals' observed interaction indexes (adjusted by total time [minutes] of observation per seal) and node-based network statistics.
* indicates significant correlation at α=0.1 level
** indicates significant correlation at α=0.05 level

	**Indexes of Observed Interaction**		
**Network Statistics**	**Count of Interactions**	**Duration of Gentle Interactions**	**Duration of Aggressive Interactions**
**Degree**	0.493*	0.678**	-0.113
**EVC**	0.488*	0.728**	-0.270
**Coreness**	0.578**	0.609**	0.060
**Closeness**	0.204	0.209	0.121
**Betweenness**	0.033	0.039	-0.055
**Transitivity**	-0.448	-0.358	-0.197

Table SI2: Comparison of association indexes and network statistics between age and sex classes of Hawaiian monk seals observed at Ka'ena Point and Rabbit Island, Oahu, Hawaii.
** indicates significant difference at α=0.05 level

**Sex**	**Age**	**n**	**Proxi-mity <5m**	**Aggressive Interact. (min.)**	**Gentle Interact. (min.)**	**Degree**	**EVC**	**Coreness**
Female	Adult	1	0.067	0.039	0.067	4.000	0.007	4.000
	Subadult	4	0.030	0.000	0.035	11.000	0.250	8.250
	Weanling	1	0.114	0.000	0.125	47.000	1.000	16.000
Male	Adult	5	0.084	0.052	0.034	11.800	0.092	8.200
	Subadult	3	0.043	0.001	0.041	10.000	0.233	6.000
Ttest Male-Female			0.550	0.078**	0.494	0.545	0.333	0.617
Ttest Adult-Subadult (males)			0.325	0.015**	0.833	0.837	0.611	0.607

Figure SI1): Social network indicating varying levels of contact among seals observed at Ka'ena Point and Rabbit Island, Oahu, Hawaii. Strength of connections is indicated by line color and thickness.

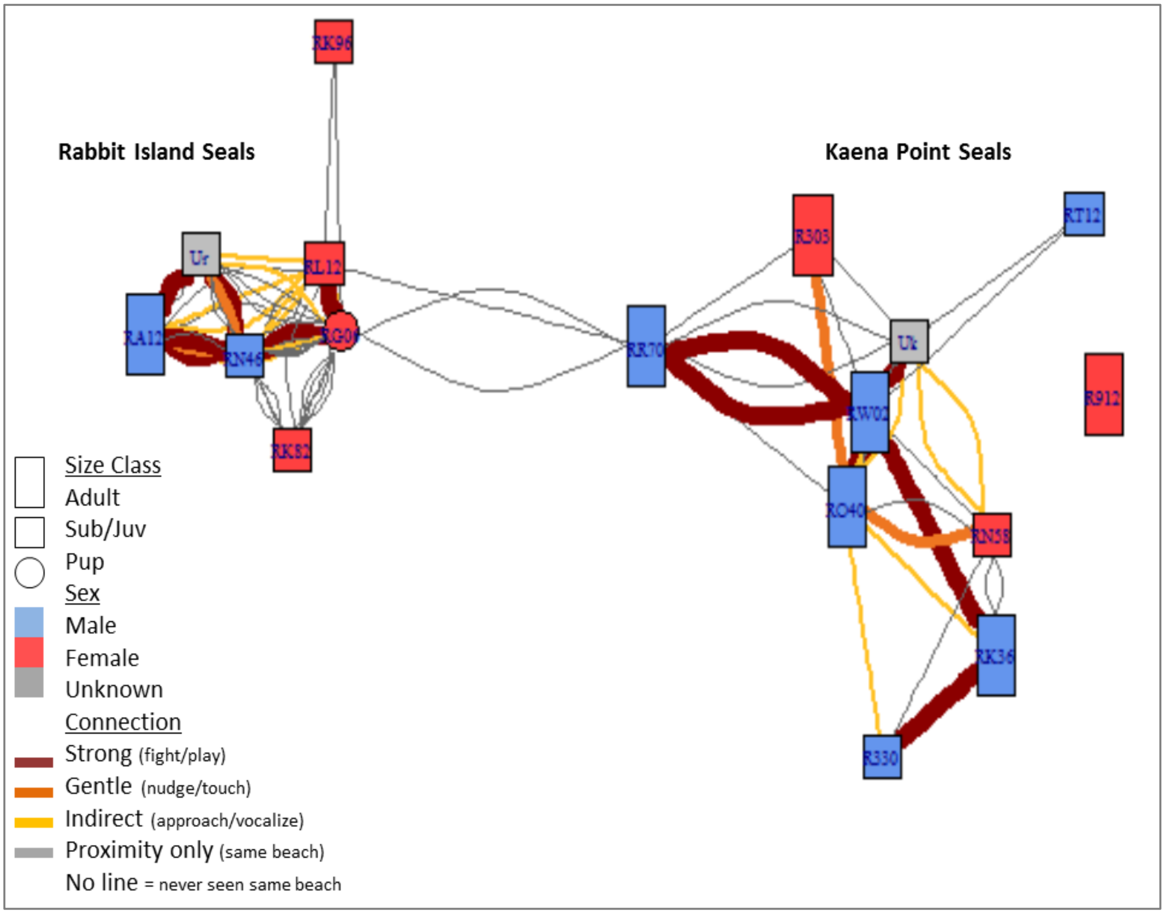

Details of Sightings Network Analysis

In order to determine whether a year of monk seal sightings data could be reasonably combined into a single representation of the population's contact structure, we constructed separate networks from the sightings from each month of 2015. We examined all 12 networks to determine whether there was month-to-month variation in the degree distribution or the specific individuals with high vs low degrees. Networks were constructed in the igraph package in R (as in the main text). Each seal was represented as a node, and each occurrence of seals ashore together on the same beach on the same day constituted a link between nodes.

There was no seasonal variation evident among the monthly networks. All monthly networks showed similar degree distributions with most seals having very few contacts, and a few seals having maximal numbers of contacts (max degree ranged from 8-12 among months; Figure SI2). Similarly, the individual seals with higher or lower degrees tended to remain consistently in the highest or lowest ranks from month to month (Table SI3). These finding provided confidence that the salient characteristics of the population contact structure could be captured in a single network summarizing sightings for the year.

Table SI3): Degree statistics from monthly seal sightings networks show the consistency with which individual Hawaiian monk seals ranked high or low in numbers of contacts observed. (Note: 0 indicates that a seal was observed in a given month, but not in contact with any other seal while a blank cell indicates that a seal was not observed in that month.)

**Seal's Overall Rank**					**Network Degree by Month**							
	**Jan**	**Feb**	**Mar**	**Apr**	**May**	**Jun**	**Jul**	**Aug**	**Sept**	**Oct**	**Nov**	**Dec**
1	11	3	3	12	10	5	7	5	0	4	6	9
2	3	2	3	8	11	4	12	9	4	7	4	2
3				3	7	3	3	7	0	0	6	9
4	1	3	1	3	6	2	3	1	3	6	4	1
5	8	6	7	7	8	6	9	9	6	5	8	3
6	9	5		1	2	5	1	4	5	5	5	9
7	6	5	8	8	6	5	7	4	7	5	4	3
8	1	4	6	8	7	9	5	4	7	8	7	8
9	3	1	6	2	0	2	1	5	3	2	2	1
10	8	8	7	8	7	6	6	9	7	8	8	7
11	9	7	4	4		5	12	7	5	5	1	5
12	0	0	4	2	1	5	4	3	2	0	3	0
13	4	5	7	8	9	6			6		4	10
14	3	3	3	2	2		5	2	1	2	0	4
15	9	5	6	3		3	8	9				
16	9	4	3	4	0	6	9	9	5	3	4	5
17	8	4		2	0	0	0	6	3	0	1	6
18				10	9	9	9	7	8	4		
19	0	0	1	6	4	1	0	1	6	5	0	2
20	6	1		2		4	4	6		4	0	
21	7	2	5	3		4	4					7
22	7	4	3	4	3	0	8	7	4	1	2	4
23	7	0	4		0	5	7	1	4	1	6	4
24	1		0	0	0	0				3	4	7
25	5	0	0	0	2	1	0		5		3	1
26				10	9		7	6				
27	0		3	4		1	4	1	0	1	0	
28	3	4	4									
29						1	4	4	2	4	5	1
30							7	4				
31	1	1	2	2	1	0		3		0	0	1
32		0						7	2			
33		1	2	4	0	0	0	1	1			1
34	0					1	4	4	2	1	2	0
35	0	0	0	1	0		0	1	1	1	1	0
36	3	1	4									
37	1	0	0	0	1	0	3			0	0	0
38										4		
39			3									
40							2					
41										1		
42							0					
43					0							
44	0	0	0			0						

Figure SI2): Histograms show the degree distribution for networks constructed from Hawaiian monk seal sightings on Oahu for each month of 2015.

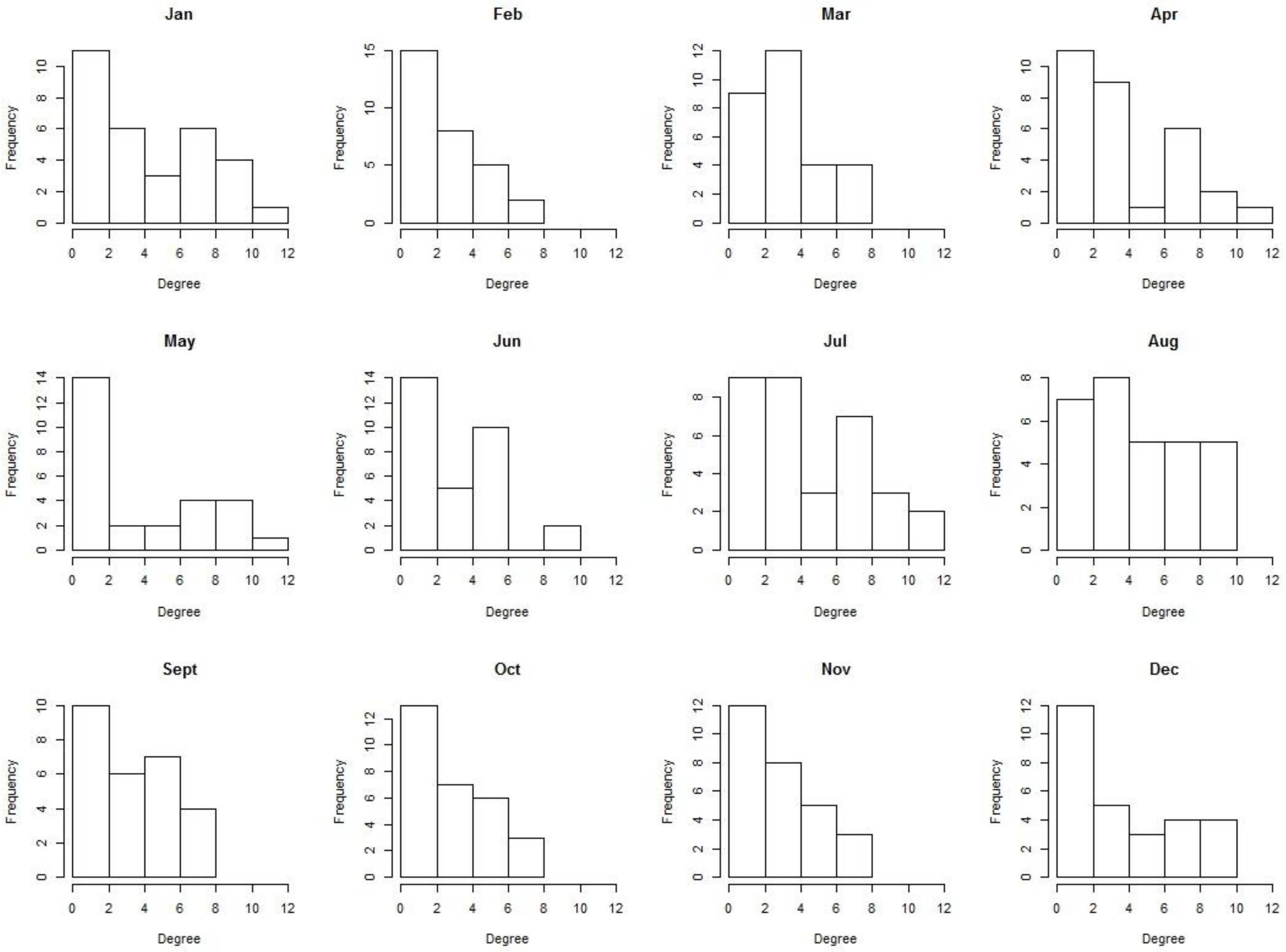

Details of parameterization for Dynamic Network Model

We used empirical contact patterns observed in the Seal Sighting Network (SSN) to parameterize the Dynamic Network Model (DNM). However, contact rates had to be rescaled because, while the SSN gave an overall view for the full year, the DNM would allow contact to form and dissolve on a daily basis. We did not simply divide the number of edges in the SSN by 365 because the contacts observed are only a survey of the total number of contacts that occur between seals in a year, and so dividing would make the daily contact rate unrealistically small. We examined the frequency of seal sighting reports on each beach in Oahu for 2015 and determined that all primary seal use areas were surveyed on a weekly basis. Thus we considered that a week's worth of sighting data represented a full-island snap shot (similar to what would be a one-day time step in the DNM where all nodes are present in each time step). Thus, we rescaled the SSN by dividing the year-long total number of contacts (edges) by 52. This was then used as the expected number of edges per time step that was used as the target value for parameterizing the DNM in EpiModel [5].

Details of parameterization for SEIR Model

We simulated a disease outbreak in Oahu's Hawaiian monk seal population using an SEIR model (Eq 1) in which individuals transition from Susceptible (S) to Exposed (E) according to the effective contact rate (β, which is the product of the rates of contact, ρ, and transmission per contact, τ) (Eq 2, 3), from E to Infectious (I) according to the length of the latency period (σ) (Eq 4), and from I to Removed (R) according to the length of the infectious period (γ) (Eq 5) after which individuals are considered 'removed' from the infectious state either through death or recovery and immunity. Thus the movement of individuals through each model compartment over time is governed by the following set of equations:

$$S \xrightarrow{\rho\tau} E \xrightarrow{\sigma} I \xrightarrow{\gamma} R \qquad \text{equation 1}$$

$$S_{t+1} = S_t - (\rho\tau)(S_t I_t) \qquad \text{equation 2}$$

$$E_{t+1} = E_t + (\rho\tau)(S_t I_t) - \sigma E_t \qquad \text{equation 3}$$

$$I_{t+1} = I_t + \sigma E - \gamma I_t \qquad \text{equation 4}$$

$$R_{t+1} = R_t + \gamma I_t \qquad \text{equation 5}$$

The parameter values entered into the above equations drive the dynamics of the simulated outbreak, thus parameterization is a critical part of examining the potential outcomes of the simulated epidemic. Because previous research has already focused on fully exploring this parameter space to predict a wide range of potential outcomes of disease in Hawaiian monk seal populations [6], here we narrowed our focus to isolate the impact of vaccination targeting (whether high vs low contact individuals were 'removed' from the susceptible pool through vaccination). However, we did explore several variations in parameters to determine how much the impact of targeted vaccination might depend on the epidemic characteristics. We implemented models with a range of parameters:

- τ: the probability of transmission given contact between an S and I was run with five variations: 0.2, 0.4, 0.6, 0.8, 1.0 (the value used in the primary analysis).
- σ: the latency rate determining the lag between exposure and infectivity (latency period in days = 1/ σ) was run with five variations: (0.035 ~ 5 days, 0.05 ~10 days, 0.07 ~14 day (the value used in the primary analysis), 0.1 ~ 20 days, 0.2 ~ 28 days).
- γ: the recovery rate determining the period in the infectious state (infectious period in days = 1/ γ) was run with five variations: (0.035 ~ 5 days, 0.05 ~10 days, 0.07 ~14 day (the value used in the primary analysis), 0.1 ~ 20 days, 0.2 ~ 28 days).

The total numbers infected in an outbreak (even without vaccination) was impacted little by the transmissibility parameter, until τ became very low. Numbers infected in the Baseline scenario only dropped from 24 – 21 as transmissibility dropped from 1.0 – to 0.4, but infections dropped to 15 at τ = 0.2 (Figure SI3A). Regardless of total numbers infected, there was a similar pattern in the performance of the targeted vaccination (Ideal scenario) vs untargeted approach (Real scenario). In all parameter scenarios, it required approximately 10 Real vaccines to lower diseases transmission to the level seen with just

5 Ideal vaccines. By the time 20 were vaccinated, infection rates were very low in general and the Real and Ideal scenarios converged.

Epidemic progression (length of the latency and infectious periods) had a larger impact on overall epidemic dynamics. Numbers infected in the Baseline scenario dropped from 28 to 5 as σ and γ varied from 0.035 (indicating a slow 28 day progression) to 0.2 (indicating a rapid progression of 5 days) (Figure SI3B). Yet, even with considerable variability in total numbers infected, the pattern in the performance of the targeted vaccination (Ideal scenario) vs untargeted approach (Real scenario) remained unchanged. Simulations still required approximately 10 Real vaccines to lower diseases transmission to the level seen with just 5 Ideal vaccines.

Table SI4): Parameters are given for each of the SEIR models run to simulate morbillivirus-like outbreaks in a monk seal population after varied vaccination scenarios. Each model was run over 100 time steps (days), and replicated in 1,000 simulations.

Epidemic Rate Parameters

**Node Set**	**Contact Rate (ρ)**		
Set1	0		
Set2	0.07		
Set3	0.11		
Set4	0.15		
Set5	0.2		
Set6	0.23		
Set7	0.26		
Set8	0.3		
Set9	0.41		
	**Transmissibility (τ)**	**Latency Period (1/σ)**	**Infectious Period (1/γ)**
**Primary analysis**	1	1/0.07	1/0.07
**Supplemental trials**	0.8	1/0.035	1/0.035
	0.6	1/0.05	1/0.05
	0.4	1/0.1	1/0.1
	0.2	1/0.2	1/0.2

Table SI4): continued

	Initial Numbers for SEIR Compartment			
Model Scenario	S	E	I	R
Baseline	44	0	1	0
Ideal1	43	0	1	1
Ideal2	42	0	1	2
Ideal3	41	0	1	3
Ideal4	40	0	1	4
Ideal5	39	0	1	5
Ideal6	38	0	1	6
Ideal7	37	0	1	7
Ideal8	36	0	1	8
Ideal9	35	0	1	9
Ideal10	34	0	1	10
Ideal11	33	0	1	11
Ideal12	32	0	1	12
Ideal13	31	0	1	13
Ideal14	30	0	1	14
Ideal15	29	0	1	15
Ideal16	28	0	1	16
Ideal17	27	0	1	17
Ideal18	26	0	1	18
Ideal19	25	0	1	19
Ideal20	24	0	1	20
Real1	43	0	1	1
Real2	42	0	1	2
Real3	41	0	1	3
Real4	40	0	1	4
Real5	39	0	1	5
Real6	38	0	1	6
Real7	37	0	1	7
Real8	36	0	1	8
Real9	35	0	1	9
Real10	34	0	1	10
Real11	33	0	1	11
Real12	32	0	1	12
Real13	31	0	1	13
Real14	30	0	1	14
Real15	29	0	1	15
Real16	28	0	1	16
Real17	27	0	1	17
Real18	26	0	1	18
Real19	25	0	1	19
Real20	24	0	1	20

Figure SI3): Epidemiological curves show the impact of variation in parameters on the outcome (numbers infected) of a morbillivirus-like outbreak simulated through an SEIR model. In A) transmissibility varies (from 1.0 to 0.2), and in B) latency and recovery rates vary (5 to 28 days). While each panel represents a different parameterization, each curve represents a different vaccination scenario. Note that, under all parameter scenarios, even when absolute numbers of infections varied, the relationship of relative infection rates among vaccination scenarios remains consistent.

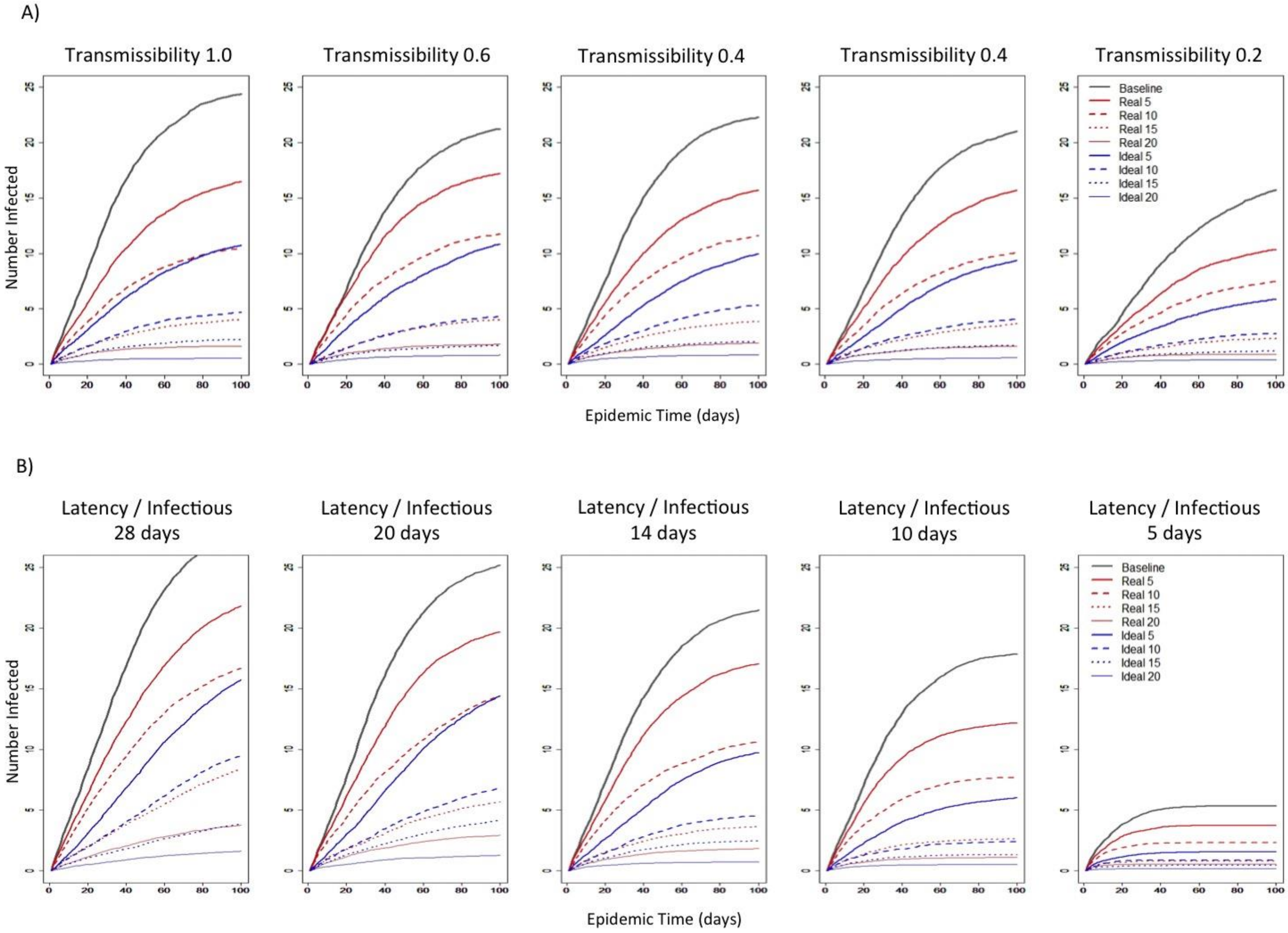

Supplemental References

1. Altmann J. 1974 Observational study of behavior: Sampling methods. *Behavior* **49**, 227-267.
2. Csardi G., Nepusz T. 2006 The igraph software package for complex network research. *InterJournal, Complex Systems*, e1695.
3. R Core Team. 2013 R: A language and environment for statistical computing. R Foundation for Statistical Computing, Vienna, Austria. URL http://www.R-project.org/.
4. Anderson B., Vongpanitlerd S. 2006 *Network analysis and synthesis: a modern systems theory approach*, Dover.
5. Jenness S.M., Goodreau S.M., Morris M. in review EpiModel: AR Package for Mathematical Modeling of Infectious Disease over Networks.
6. Baker J.D., Harting A.L., Barbieri M.M., Robinson S.J., Gulland F.M., Littnan C.L. 2017 Modeling a morbillivirus outbreak in Hawaiian monk seals to aid in the design of mitigation programs. *Journal of Wildlife Diseases*, online early.
